# Effects of Hydrochar Incorporation on the Nitrogen Leaching Flux Pattern and Load in Rice Paddy Soil and Crop Production

**DOI:** 10.3390/plants14030455

**Published:** 2025-02-04

**Authors:** Meng Ma, Yudong Chen, Jinjin Zhang, Chang Liu, Haijun Sun

**Affiliations:** 1 Co-Innovation Center for Sustainable Forestry in Southern China, College of Forestry and Grassland, Nanjing Forestry University, Nanjing 210037, China; mameng@njfu.edu.cn (M.M.); liuchang@njfu.edu.cn (C.L.); 2 State Environmental Protection Key Laboratory of Soil Environmental Management and Pollution Control, Nanjing Institute of Environmental Sciences, Ministry of Ecology and Environment of China, Nanjing 210042, China; 3 Office of Educational Administration, Linyi Vocational University of Science and Technology, Linyi 276025, China; lingyunmaomao@163.com

**Keywords:** crop production, hydrochar, N fertilizer, N leaching, rice yield

## Abstract

Hydrochar (HC) incorporation affects soil nitrogen (N) transformation, which could further affect the N leaching loss. We conducted a soil lysimeter experiment to evaluate the responses in terms of N leaching and rice yield to HC applied at a low (0.5%) or high (1.5%) rate, while considering three N inputs, i.e., 240, 192, and 144 kg/ha (named N240, N192, and N144, respectively). The results showed that the rice grain yield was highest (124.3 g/pot) for N192, while being significantly reduced to the minimum yield achieved in the study (110.3 g/pot) for N144. Interestingly, for the N input 144 kg/ha, HC application increased the rice grain yield by 6.9–8.0%, which was equivalent to that of N240. NH_4_^+^-N leaching occurred mainly during the first 4 weeks of the rice season, and HC did not influence NH_4_^+^-N leaching for both the N inputs, 192 and 240 kg/ha. However, compared to N144, N144 + HC1.5% recorded a significantly higher NH_4_^+^-N leaching loss of 34.6%. This suggests that the application of a high amount of HC increases the NH_4_^+^-N leaching risk when the N input is low. HC application resulted in 10.2–45.3% more NO_3_^−^-N leaching loss when the three N inputs were applied, the effect of which was significant in regard to the applications involving a 20 and 40% N reduction, but this occurred only with the applied treatments involving 1.5% HC. Moreover, we found that organic N was the main form of leachate N (>80%). More specifically, N144 + HC recorded 7.8–8.3% lower organic N leaching than N192. Based on the effects of HC on the rice grain yield and N leaching, we recommend applications involving a 40% N reduction (N144) with a lower amount of HC (HC 0.5%) to ensure high crop production and to protect the water environment.

## 1. Introduction

Nitrogen (N) is the main limiting nutrient in regard to crop growth and development, and N fertilizer can increase crop yields [1]. Therefore, applying N fertilizer is an important measure to ensure stable and high rice yields [2]. However, in pursuit of higher rice yields, farmers often apply excessive N fertilizer inputs in actual production [3]. Previous studies have demonstrated that the average utilization rate of N fertilizer in rice production is about 30% and is even lower in some high-yielding areas [4]. High application amounts but low uptake rates result in most of the N fertilizer being retained in the soil and a small proportion being taken up by the crop, leading to excess soil nutrients [5]. Consequently, a large quantity of the excessive N is lost from the soil–plant system via ammonia and nitrous oxide emissions, as well as leaching [6], which lead to global climate change and the eutrophication of rivers and lakes [7,8]. In this context, N leaches into water bodies in the form of ammonium (NH_4_^+^-N), nitrate (NO_3_^−^-N), and organic N, which can pollute groundwater. Furthermore, after being reduced to NO_2_^−^-N, NO_3_^−^-N threatens human health by causing diseases, such as methemoglobinemia and cancers of the digestive system [9]. The threat from NO_3_^−^-N contamination is more serious in countries and regions where groundwater is the main source of drinking water [10]. It is evident that N leaching from paddy fields needs to be given serious attention and that the quantity of N fertilizer applied during crop production needs to be closely regulated. Thus, how to maintain rice productivity, while applying less N fertilizer, becomes an important issue for landowners and the authorities. In addition to conventional nutrient management techniques like precise fertilization, scientific crop rotation, and the rational use of organic fertilizers [11], applying appropriate soil additives helps to improve the rice yield and N use efficiency, thereby reducing N leaching losses from paddy fields [12].

In recent years, the use of biochar (BC) as a soil additive has attracted widespread attention, in particular research has been carried out to evaluate its effects on the N environment [13,14]. According to the differences in the preparation process, BC can be divided into two categories: pyrochar and hydrochar (HC). Previous research has focused on the agricultural utilization of pyrochar that was produced in high-temperature, oxygen-limited conditions [15,16]. Unlike pyrochar, HC is produced in an airtight container, subject to autogenous pressure and a relatively moderate temperature (150–375 °C), with crude material and liquid as the solvent and medium, respectively [17]. In addition to its well-developed pore structure, high carbon content, and unique functional groups like conventional pyrochar [18], HC can retain more organic matter and nutrients (N, phosphorus, and others) and has richer oxygen and N-containing groups, all of which slow down the nutrient release rates and their leaching into the soil [19]. Therefore, HC also has potential as a soil conditioner for agricultural and environmental applications. Compared to other aspects related to the effects of HC, such as waste biomass resourcing [20] and heavy metal sequestration [21], etc., few studies have evaluated the effects of HC on N leaching from agriculture soil. Bargmann et al. [22] found that HC acted as a slow-release fertilizer to promote the growth and biomass of barley and kidney beans. This suggests that it is feasible to ensure a high crop yield through reduced N fertilizer application after adding HC. Takaya et al. [23] and Cai et al. [24] both showed that HC has good N adsorption capacity. In addition to decreasing N leaching due to a reduction in the application of N fertilizer, it can be hypothesized that the addition of HC has the potential to further reduce N leaching losses from paddy fields. Jandl et al. [25] noted that HC tends to be weakly acidic in most preparation conditions and often carries many organic compounds (organic acids, organic phenols, etc.) on its surface, which may affect soil N transformation and, consequently, N leaching and crop growth. For instance, Poerschmann et al. [26] found that the direct application of HC at a high rate to paddy fields decreased the crop yields. Therefore, further research is needed to determine the appropriate application rate of HC in practical applications, with a view to enabling HC to exert its positive effects in regard to soil improvements, crop yield increases, and N loss reductions.

Generally, how HC, applied at varied rates, impacts crop yields and N leaching from paddy soils in reduced N fertilizer conditions is not fully understood. In this study, we conducted a soil column experiment to clarify the aforementioned effects of HC after being incorporated into rice paddy soil. The results will provide a theoretical basis and reference data for the rational application of HC to realize N use efficiency improvements and leaching loss mitigation, which are beneficial for the paddy field environment.

## 2. Results

### 2.1. Rice Yield

Table 1 indicates that there was no significant change in the rice straw biomass following either a reduction in the application of N or the application of HC. The N192 treatment produced the highest rice grain yield (124.3 g/pot), while the N144 treatment (40% N reduction) produced a significantly (*p* < 0.05) lower rice grain yield of 11.3%. The rice grain yield was not affected by the applications of 0.5% and 1.5% HC when both a conventional N application and a 20% N reduction were applied. Interestingly, in regard to the 144 kg N ha^−1^ treatment, the HC addition increased the rice grain yield by 6.9–8.0%, equivalent to that of medium and high N application treatments. In terms of yield component factors, this was mainly characterized by an increase in the number of spikes per pot (11.1–18.5%). Comparing the 20% N reduction to conventional N application circumstances, it is evident that there was no significant change in rice production; the rice grain yield can be guaranteed by increasing the N reduction up to 40%, coupled with a lower quantity of HC.

### 2.2. Nitrogen Uptake by Rice Plants

In regard to the conventional N treatments applied, both HC0.5% and HC1.5% did not affect the N uptake capacity of rice straw (Table 2). However, there was a positive correlation between the HC application rate and the rice straw total N uptake for the 192 kg/ha N input (20% N reduction). In particular, we found that when comparing N192 + HC1.5% to N192, the rice straw total N uptake capacity increased from 2.2 mg/pot to 6.0 mg/pot. However, when the N fertilizer was reduced further to 144 kg/ha, HC increased the rice straw total N and the N uptake of the rice straw when applied at the 0.5% rate, but decreased it when applied at the 1.5% rate. Consistently, it is evident that the total N uptake amounts of the rice straw were significantly (*p* < 0.05) increased following the addition of HC at the 0.5% rate for both the 20% and 40% N fertilizer reductions.

As demonstrated in Table 2, N uptake by rice grains and the total N uptake were not significantly affected by the N reduction or HC application. In regard to the conventional N application and 20% N reduction, there was no significant impact on the total N and N uptake of the rice straw; however, when the N reduction rate was increased to 40%, both the total N and N uptake of the rice straw showed a significant increase of more than 50% (*p* < 0.05).

### 2.3. Effects of HC on N Leaching

#### 2.3.1. NH_4_^+^-N

**Figure 1:**
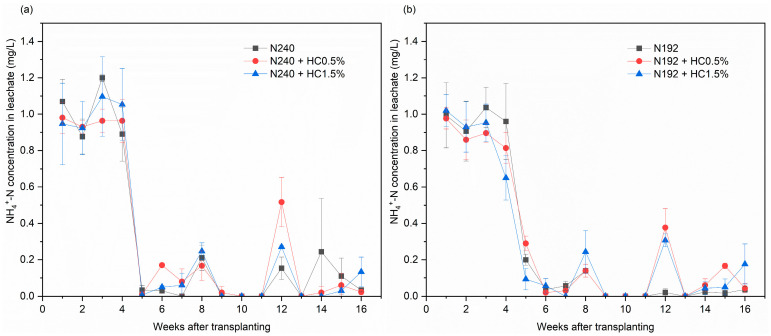

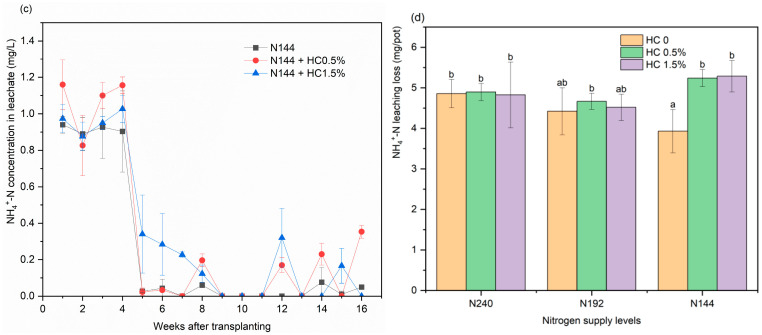
Effects of applying different amounts of HC on the NH_4_^+^-N concentration in leachate for N240 (**a**), N192 (**b**), N144 (**c**) input treatments and on the total NH_4_^+^-N leaching losses (**d**). Data are shown as mean ± SD (*n* = 3). Different lowercase letters above the column indicate significant differences between treatments, according to Duncan’s multiple range test (*p* < 0.05).

For all the treatments, the leachate NH_4_^+^-N concentration showed a generally declining trend during the rice growth cycle observations (Figure 1a–c). For the first 4 weeks, the leachate NH_4_^+^-N concentration was relatively high, from 0.7 to 1.2 mg/L, and, thereafter, dropped very quickly, to almost the no detection level. Overall, for the observations after the 5th week, the leachate NH_4_^+^-N concentration varied little, at a low level that was below 0.52 mg/L. The data in Figure 1 also suggest that the N reduction or the HC application did not change the fluctuation pattern of leachate NH_4_^+^-N.

When no HC was added, the NH_4_^+^-N leaching losses decreased with the reduction in the N fertilizer application. The data in Figure 1d shows that the result for N144 was significant (*p* < 0.05), with a 19.1% lower NH_4_^+^-N leaching loss than N240. The HC0.5% and HC1.5% applications did not affect NH_4_^+^-N leaching for both the conventional N application and the 20% N reduction. In contrast, when the N reduction was increased to 40%, the application of HC resulted in a significant increase in NH_4_^+^-N leaching losses, by almost 35% (*p* < 0.05). This demonstrates that the application of HC with the 40% N reduction increased the NH_4_^+^-N leaching risk.

#### 2.3.2. NO_3_^−^-N

**Figure 2:**
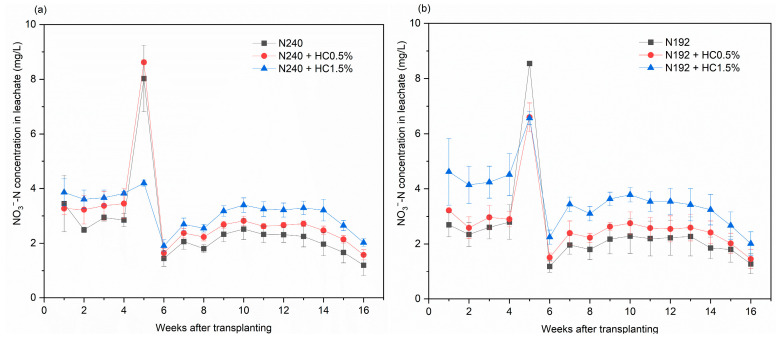

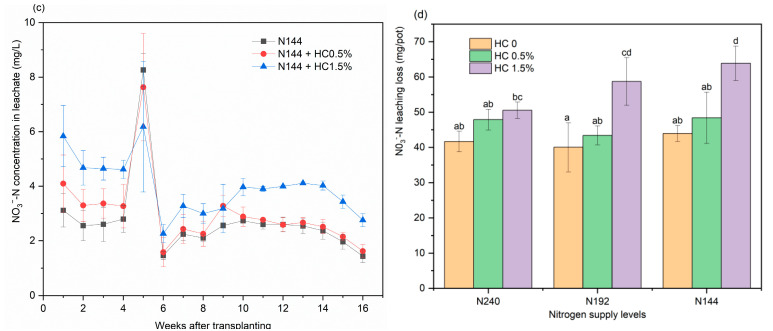
Effects of applying different amounts of HC on the NO_3_^−^-N concentration in leachate for N240 (**a**), N192 (**b**), N144 (**c**) input treatments and on the total NO_3_^−^-N leaching losses (**d**). Data are shown as mean ± SD (*n* = 3). Different lowercase letters above the column indicate significant differences between treatments, according to Duncan’s multiple range test (*p* < 0.05).

A similar trend was observed in regard to the changes in the leachate NO_3_^−^-N concentration during the rice season (Figure 2a–c). The leachate NO_3_^−^-N concentration fluctuated within a low range of 2.4–5.8 mg/L in the first 4 weeks. It sharply increased to the maximum value for each treatment (4.2–8.6 mg/L) during the 5th week. Thereafter, the leachate NO_3_^−^-N concentration rapidly declined to only 1.44 mg/L in the 6th week and, thereafter, it fluctuated between 1.2 and 4.1 mg/L until the 10th week. Thereafter, the NO_3_^−^-N concentration in the leachate for all the treatments showed a declining trend, along with the rice-growing period. We also noted that, except for the observation at the 5th week, the leachate NO_3_^−^-N concentration showed the pattern HC1.5% > HC0.5% > no HC treatment and that this pattern was independent of the N input rate.

There was no significant correlation between the N input rate and the N loss via NO_3_^−^-N leaching, the losses of which ranged from 40.0 to 43.9 mg/pot for the three treatments that did not include the addition of HC (Figure 2d). Nevertheless, HC addition increased the NO_3_^−^-N leaching losses by 15.0–21.3%, 8.4–46.6%, and 10.2–45.3% for the conventional, 20%, and 40% N reduction treatments. In particular, for the 20–40% N reduction conditions, the effect of HC1.5% on the increase in NO_3_^−^-N leaching losses reached a significant level (*p* < 0.05).

#### 2.3.3. Organic N

**Figure 3:**
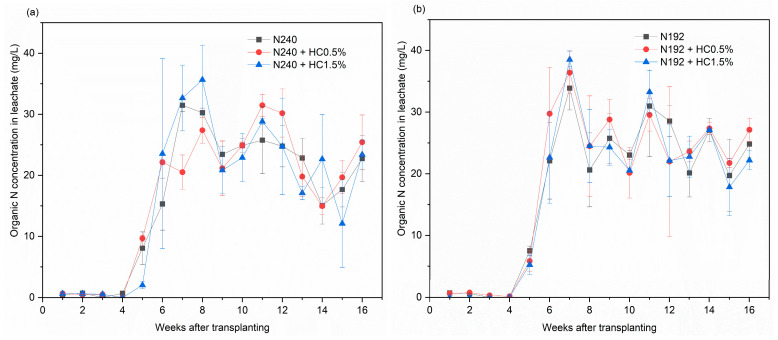

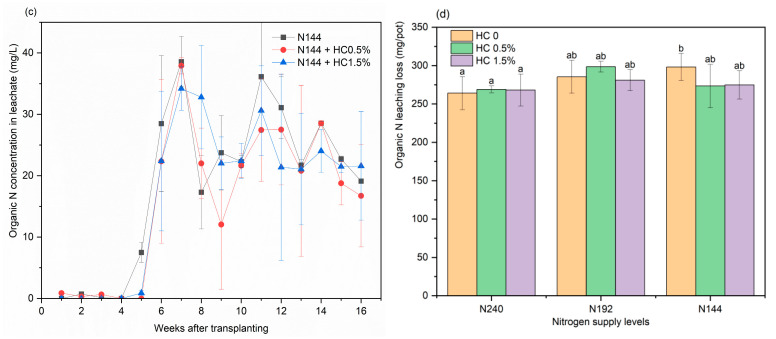
Effects of applying different dosages of HC on the organic N concentration in leachate for N240 (**a**), N192 (**b**), N144 (**c**) input treatments and on the total organic N leaching losses (**d**). Data are shown as mean ± SD (*n* = 3). Different lowercase letters above the column indicate significant differences between treatments, according to Duncan’s multiple range test (*p* < 0.05).

For three N inputs, the changing patterns in the leachate organic N concentration were similar (Figure 3a–c). The organic N concentrations were stably below 0.9 mg/L during the first 4 weeks and, thereafter, they dramatically increased during weeks 5–7 and showed a maximum value in the 7th week (20.5–38.6 mg/L) for most treatments (Figure 3a–c). During the 9–16th weeks, the concentration of organic N in the leachate varied significantly between 12.1 and 36.1 mg/L, and no consistent flux pattern could be seen during this period.

The data in Figure 3d show that the highest organic N leaching loss occurred with a value of 298.1 mg/pot for the N144 treatment. Compared with the N240 treatment, however, the 20% N reduction treatment (N192) had no significant effect on the organic N leaching loss. For all three N supply levels, the HC addition did not significantly affect the amount of organic N leaching loss.

#### 2.3.4. Total N Leaching

The varying pattern in the total N concentration in the leachate from all the treatments was consistent. For the first 4 weeks, the total N concentration in the leachate fluctuated within the low range of 3.6–6.3 mg/L. During weeks 4–7, the total N leaching concentration for most treatments increased rapidly, reaching a peak in the 7th week, wherein the maximum values were recorded (33.6–42.0 mg/L). Only two treatments involving N240 with additional HC peaked in week 8 (29.8 and 38.5 mg/L). During weeks 9–16, the total N concentration in the leachate varied widely for almost all the treatments, except for three treatments involving N144, which exhibited a declining trend. The remaining six treatments involving N240 and N192 varied from 14.8 to 38.7 mg/L, and no coherent flow pattern could be identified during this period.

**Figure 4:**
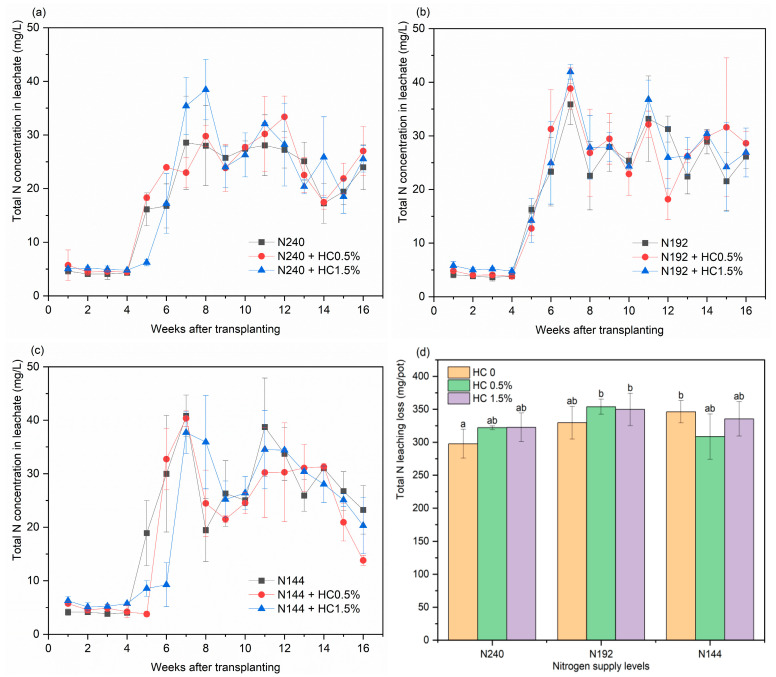
Effects of applying different dosages of HC on the total N concentration in the leachate for N240 (**a**), N192 (**b**), N144 (**c**) input treatments and on the total N losses (**d**). Data are shown as mean ± SD (*n* = 3). Different lowercase letters above the column indicate significant differences between treatments, according to Duncan’s multiple range test (*p* < 0.05).

Like organic N leaching losses, comparing N144 to N240, when the N reduction rate was increased to 40%, the total N leaching losses were significantly elevated by 16.3% (*p* < 0.05) (Figure 4d). The total N leaching loss was not significantly affected by the addition of HC in regard to any of the three N supply levels. Regarding the independent N supply level, HC increased the total N leaching loss for the N240 and N192 treatments. In contrast, after increasing the reduction to the 40% N reduction, the addition of HC resulted in a decrease in the total N leaching loss; HC0.5% showed a larger decrease of 8.9% than HC1.5%, which was just 10.0 mg/pot above the minimum value (297.7 mg/pot). It is evident that a 40% N reduction coupled with a low amount of HC can effectively reduce the total N leaching loss.

## 3. Discussion

### 3.1. Rice Growth and Crop Yield Response to Hydrochar

Previous studies have demonstrated that reducing the amount of N fertilizer applied by a reasonable rate does not lead to a crop yield reduction [27,28], which was also confirmed by our data. Interestingly, we found that the rice grain yield from the N192 (with 20% N reduction) treatment was even higher than that from the conventional N treatment. This result suggests that soil fertility in 20% N reduction conditions is rich and, at this point, additional N fertilizer to increase the crop yield is not necessary because a 20% reduction in N fertilizer can provide enough N necessary for rice growth and grain yield. However, the rice grain yield significantly decreased when the N reduction was reduced further to 40%, as a result of the insufficient N supply. Therefore, too large a N reduction, e.g., ≥40%, as shown in the current work, was not reasonable, considering the crop production needs. Interestingly, the addition of HC could make up for the negative effect caused by the 40% N reduction on the rice yield. This might be the result of the available nutrients carried and released by the HC itself [29]. What is more, HC’s porous properties improves the physicochemical characteristics of the soil [30,31] and, thereby, its capacity to supply nutrients [32], all of which helps to increase rice grain yields. Moreover, Yu et al. [33] and Ji et al. [29] confirmed the positive effect of HC on increasing the crop yield. Of course, there must be some nutrients that are contributed by the HC itself that can enhance crop growth, which should be studied in future.

However, HC combined with the N192 treatment resulted in a decrease in the rice grain yield, which also happened in regard to the N144 + HC1.5% treatment, the effect of which was different from that observed in regard to the N144 + HC0.5% treatment. This could be due to imbalances in the soil C/N ratio caused by the addition of HC [34], which limited the N absorption capacity of the rice plants. These results suggest that HC should be applied at an appropriate rate and combined with reasonable N inputs. Previously, Zhou et al. [35] and Bona et al. [36] reported a detrimental impact of direct HC application on crop plant growth and this negative effect was more pronounced when the HC was applied in higher amounts. Furthermore, Purakayastha et al. [37] found that the actual effect of HC on crop yield and N utilization depends primarily on the type of HC used, such as that derived from different feedstock and pyrolysis temperatures. In addition,, we found that the response in terms of crop productivity to HC was dependent on the HC and N input amounts.

### 3.2. The Responses of N Leaching to Hydrochar Applied into Paddy Soil

Most of the N that leaches from the soil is organic N (>80%), followed by NO_3_^−^-N and NH_4_^+^-N. By investigating the distribution of organic N in the soil, Nie et al. [38] also found that the estimated annual organic N leaching losses from rice paddies accounted for more than 50% of the total N leaching losses. Similar variations were seen in the organic and total N concentrations in the leachate in this study, with most of the leaching taking place in weeks 5–16 after the paddy fields were dried by the sunshine. For varying amounts of N application, there were variations in the organic and total N concentrations in the leachate. Compared to the N240 treatment, a significant increase in organic N leaching occurred for the N144 treatment (*p* < 0.05). The observed phenomenon may have its origins in the notable decline in the rice yield during periods of low N supply. This decline in yield could have, in turn, caused a reduction in rice root secretion and its ability to promote the conversion of organic N into inorganic N [39], which would have increased the leaching of organic N. The HC addition did not significantly affect the amount of organic N losses for the three N supply levels. Interestingly, compared with the N144 treatment, the addition of HC resulted in a decrease in the amount of organic N leaching loss, which appeared to be lower than that for the mid-applied N treatment. It is evident that the amount of organic N leaching losses can be effectively reduced by applying HC when a low level of N has been applied. The decrease in organic N leaching after its application may be explained by the ability of HC to assist the conversion of organic N into inorganic N. Xu et al. [40] found that HC enhanced the soil’s bacterial diversity and microbial biomass significantly, which may be one of the factors driving the conversion of organic N. This is corroborated by the decrease in organic N leaching and the increase in NH_4_^+^-N and NO_3_^−^-N (inorganic N) leaching losses due to the application of HC, in comparison to low N supply treatments.

In this experiment, for the three levels of N supply, there was no significant effect of HC on the trend in terms of the soil NH_4_^+^-N and NO_3_^−^-N leaching loss. The application of basal and tiller fertilizers may have contributed to the NH_4_^+^-N leaching that occurred mainly in the first 4 weeks. After the drying of the paddy fields in the sunshine, the leaching quickly decreased and oscillated at a low level during the remaining growth period of the rice. The NO_3_^−^-N concentration in the leachate remained mostly steady, peaking only in the 5th week due to the drying of the paddy fields in the sunshine. The NO_3_^−^-N concentration then showed a sharp decrease in the 6th week, which was lower than that in weeks 1–4, and then fluctuated within a narrow range and showed a decreasing trend in the later period. In general, the trend in the inorganic N concentration in the leachate during the whole rice growth period was in line with previous studies [41,42]. The total NH_4_^+^-N leaching loss was not substantially affected by the HC addition for either the 20% N fertilizer reduction treatment or the conventional N application; however, NH_4_^+^-N leaching from the soil was significantly increased (*p* < 0.05) when the 40% N fertilizer reduction was maintained. In a similar vein, adding HC led to higher NO_3_^−^-N leaching losses for all three N application levels; for the 20–40% N decrease, the incremental NO_3_^−^-N leaching losses reached significant levels (*p* < 0.05) with the HC1.5% application. Therefore, it is reasonable to infer that adding HC to the soil could result in higher NO_3_^−^-N leaching losses and that there is a positive association between the amount of leaching and the HC application amount. This might be the result of adding HC particles to the soil, which increase soil porosity and increase the overall amount of leachate, increasing the amount of NH_4_^+^-N and NO_3_^−^-N leaching. Moreover, HC’s limited specific surface area could be the reason for its poor NH_4_^+^ adsorption ability [43]. In addition, HC may only be able to absorb trace quantities of NO_3_^−^-N due to their poor anion exchange capacity, which rapidly diminishes when it is oxidized in soil. It has also been discovered that the type of HC and the N morphology have an impact on how NH_4_^+^-N and NO_3_^−^-N are retained and released [41,44]. Yao et al. [45] found that most biochars showed little to no potential for NO_3_^−^-N adsorption after testing 13 biochars (including HC). As a result, applying too much HC runs the risk of promoting NH_4_^+^-N and NO_3_^−^-N (especially NO_3_^−^-N) leaching.

The HC was not completely oxidized at the end of the experiment, since the soil column test was conducted over a short period. According to Singh et al. [46], the oxidation of the HC surface with longer application times may boost the adsorption potential of HC. Further investigation is required to ascertain whether the nutrient adsorption capabilities of HC can be improved with age. Furthermore, Zhang et al. [47] demonstrated that adsorbing N fertilizer into biochar and then mixing it with soil reduced the N loss more effectively than mixing soil, biochar, and N fertilizer all at once, and that HC may also possess this quality. Additionally, the study by Hou et al. [48] also demonstrated that applying HC to farmland after modification can increase crop yields and reduce N losses from farmland into the environment. In order to enhance biocompatibility and avoid negative impacts on the crop-growing environment, the rate at which HC is applied to agricultural production should be carefully controlled or altered.

## 4. Materials and Methods

### 4.1. Experimental Materials

As a common type of agricultural biowaste, the discarded leaves of Chinese cabbage (*Brassica oleracea* L.) were used as raw material for HC production. The reaction temperature and pressure in a high-pressure hydrothermal reactor were 260 °C and 8 MPa, respectively. After 1.5 h, the product was filtered through non-woven fabrics and then soaked in deionized water for 1 h, stirred for 30 min, filtered, and dried for use in the current experiment [49]. The basal physicochemical properties of the HC were: pH 4.7, C content 44.1%, N content 2.3%, and ash content 26.7%. The HC pH was measured in a 1:5 (*w*/*v*) HC/water suspension, using a combined reference electrode (Φ255 pH/temp/mV meter). The total C was determined using an Elemental Analyzer (PE 2400 Series II CHNS/O Elemental Analyzer, PerkinElmer, Inc., Waltham, MA, USA). After H_2_SO_4_–H_2_O_2_ oxidation, the N was measured using the Kjeldahl method [35,49]. The ash content was determined by measuring the weight loss of the hydrochar in a muffle furnace at 750 °C for 3 h [50].

The rice selected for the experiment was *Oryza sativa* L., var. Nangeng 46, which is widely grown in the Taihu Lake area of China, and soil was collected from Zhoutie Town, Yixing City (31°28′ N, 119°59′ E), Jiangsu Province. This region has a subtropical monsoon climate, with an average annual temperature and rainfall of 16.1 °C and 1210 mm, respectively. The soil samples were collected according to the subsections of 0–20, 20–40, and 40–60 cm in profile and then they were air dried for about 10 days. After being sieved through a 2 mm sieve, a total of 35 kg of soil samples was repacked into each soil column, with the same bulk density (1.3 g/cm^3^) as that of field conditions. The soil column, with an inner diameter of 35 cm and a height of 70 cm, was made of polyvinyl chloride material. The tested soil was classified as paddy soil and the selected properties of the 0–20 cm of topsoil were as follows: pH 6.4 (soil–water ratio 1:5), soil organic matter 29.2 g/kg, total N 1.7 g/kg, available P 23.1 mg/kg, and available K 159.3 mg/kg. The soil analysis was conducted as a preliminary experiment in this study, which was mainly used to provide the basic properties of the tested topsoil. The soil pH was measured using the same method as for the HC. The potassium dichromate oxidation heating method was used to determine the soil organic matter (SOM). The total N content was measured using the Kjeldahl method. The soil’s available P content was measured using a molybdenum antimony antichromogenic agent and spectrophotometry. The soil’s available K content was determined using NH_4_^+^-N acetate extraction–flame photometry [51].

### 4.2. Experimental Design

Nitrogen fertilizer (urea) was applied at three rates, i.e., the farmers’ conventional N rate 240 kg/ha (N240), and two scientifically recommended N reductions of 192 kg/ha (N192) and 144 kg/ha (N144). For each N input rate, HC was applied at ratios of 0.5% *w*/*w* and 1.5% *w*/*w*, based on the dry weight of the first 0–20 cm of topsoil (10 kg), respectively. Therefore, a total of nine treatments were labelled as N240, N240 + HC0.5%, N240 + HC1.5%, N192, N192+ HC0.5%, N192+ HC1.5%, N144, N144+ HC0.5% and N144+ HC1.5%. Each treatment was replicated three times, therefore there was a total of 27 soil columns. We evenly incorporated the weighted HC into the first 0–20 cm of topsoil at the initiation of the experiment.

The rice was transplanted into the soil columns, with a density of 6 seedlings (28 days old) per soil column. Urea (46% N), calcium superphosphate (12% P_2_O_5_), and potassium chloride (60% K_2_O) were used to supply the N, P, and K nutrients for the rice, respectively. Urea fertilizer was applied three times, with a ratio of 4:3:3, including application times for the base fertilization, tiller fertilization, and panicle fertilization, and the application periods were 2 July, 22 July, and 31 August in 2023. Both the phosphate and potassium fertilizers were applied as basal fertilizers to all the treatments at rates of 96 kg/ha P_2_O_5_ and 192 kg/ha K_2_O in the form of calcium superphosphate and potassium chloride, respectively. The floodwater was drained during a mid-season drainage period from 1 August to 7 August 2023 to control invalid tillering and improve the development of the rice plant roots. During the other times, a floodwater depth of 3–5 cm was maintained with tap water. Weeds and pests were controlled according to the traditional practices of local farmers.

### 4.3. Sample Collection and Measurement Method

#### 4.3.1. Leachate Samples

In this experiment, drainage holes, pipes, and valves were designed and installed at the bottom of the soil columns for weekly leachate sample collection (7.10, 7.17, 7.24, 7.31, 8.7, 8.14, 8.21, 8.28, 9.4, 9.11, 9.18, 9.25, 10.02, 10.09, 10.18, and 10.23) during the rice growth cycle. The average leaching rate in a rice paddy field in the Taihu Lake area was approx. 2 mm/d [52], according to which we collected 1000 mL of water samples per column at each observation. And then the water samples were filtered into clean plastic bottles to be stored at −20 °C until further analysis. The total N concentration in the leachate was determined using ultraviolet spectrophotometry, based on absorbance [53]. The concentrations of inorganic nitrogen (NH_4_^+^-N, NO_3_^−^-N) in the leachate were determined using an autoanalyzer (SKALAR San++ System, Skalar Analytical B.V., Breda, The Netherlands). The presence of organic N was determined using the difference subtraction method, based on the difference between the total nitrogen content and the inorganic nitrogen content. The cumulative N leaching loss for each indicator, namely NH_4_^+^-N, NO_3_^−^-N, organic N, and total N, was the sum of the weekly leaching loss in terms of that indicator during the experiment.

#### 4.3.2. Plant Samples

Rice straw and grains were separately harvested on 7 November 2023. Thereafter, we measured the rice plant height and recorded the yield-related agronomic traits, including the number of panicles, number of grains per panicle, and 1000 seed weight. Parts of the straw and grain samples were oven dried at 105 °C for 30 min, then dried at 80 °C until a constant weight was achieved. The dried plant samples were ground into powder using a high-speed crusher (FW100, Tianjin Taisite Instrument Co., Ltd., Tianjin, China) and passed through a 0.20 mm nylon sieve. The ground plant samples were kept in sealed containers until digestion. A sub-sample (0.25 g) of the ground plant samples were digested in a mixture of H_2_SO_4_ and H_2_O_2_ and used for the determination of the total N content, using the Kjeldahl method [51]. The nitrogen uptake by the straw and grain was calculated according to the following equation:

$$NU=\frac{m\times c}{1000}$$

where NU is the straw (grain) N uptake (mg/pot); m is the straw (grain) dry weight (g/pot); and c is the total N content (%) of the straw (grain).

### 4.4. Data Processing and Statistical Analysis

All the statistical analyses were performed using Microsoft Excel 2010 and SPSS 27.0 statistical software. The differences among the nine treatments were analyzed using a one-way analysis of variance (ANOVA), and all the tests in terms of the significance of such differences among the treatments were conducted using Duncan’s multiple-comparison test (*p* < 0.05). Data plotting was performed using Origin 8.0 and Microsoft Excel 2010 software.

## 5. Conclusions

We conducted soil column experiments to investigate the effects of HC application, for two different amounts, on neutral soil N leaching losses and rice production, coupled with a reduction in N fertilizer input. In regard to the conventional N input, the HC addition did not significantly affect the rice yield. The 20% N reduction treatment produced the highest rice grain yield, whereas the 40% N reduction treatment significantly decreased the rice grain yield. Interestingly, for the 40% N reduction, the HC addition was able to increase the rice grain yield to the level of that achieved by the medium and high N application treatments. The HC addition showed no remarkable impact on NH_4_^+^-N leaching for the 240 kg/ha treatment. However, when the N application was reduced to 192 and 144 kg/ha, the HC addition presented the risk of increasing the NH_4_^+^-N leaching loss. For all three N application levels, the addition of HC led to relatively higher NO_3_^−^-N leaching losses, with notable increases in NO_3_^−^-N leaching losses observed for the 20–40% N reductions with HC1.5% applications. Since soil N was mainly leached in the form of organic N and N144 + HC effectively reduced the organic N leaching loss to an even lower level than that of the N192 treatment, N144 + HC0.5% was found to have the lowest total N leaching loss among all the treatments. In conclusion, it is recommended that HC should be applied at a low rate and in reduced N conditions (e.g., 0.5% HC and 40% N reduction, respectively, in the current work), considering the crop yield and N environment. Otherwise, there is a potential risk of increasing N leaching following the application of HC.

## Figures and Tables

**Table 1:** The effect of applying different amounts of hydrochar (HC) with different nitrogen supply levels on rice yield and its constituent factors.

Treatment	Straw Biomass	Grain Yield	Yield Components		
	(g/pot)	(g/pot)	Spike No. per Pot	Kernel Number per Spike	Thousand Kernel Weights (g)
N240	113.10 ± 16.86 a	117.61 ± 11.33 ab	30 ± 1 b	147 ± 13 a	27.46 ± 0.85 a
N240 + HC0.5%	105.84 ± 10.91 a	117.56 ± 8.94 ab	30 ± 1 b	145 ± 14 a	27.03 ± 0.14 a
N240 + HC1.5%	108.58 ± 4.05 a	121.75 ± 2.41 ab	29 ± 1 ab	149 ± 8 a	27.48 ± 0.33 a
N192	115.64 ± 11.05 a	124.31 ± 6.52 b	30 ± 2 ab	151 ± 4 a	27.96 ± 0.69 ab
N192 + HC0.5%	113.38 ± 6.91 a	116.18 ± 6.20 ab	29 ± 3 ab	146 ± 6 a	27.49 ± 1.00 a
N192 + HC1.5%	115.24 ± 7.33 a	121.66 ± 6.35 ab	31 ± 2 b	147 ± 11 a	27.99 ± 1.04 ab
N144	101.36 ± 2.93 a	110.30 ± 4.80 a	27 ± 2 a	153 ± 7 a	27.55 ± 0.27 a
N144 + HC0.5%	109.90 ± 6.60 a	119.11 ± 6.30 ab	32 ± 3 b	142 ± 11 a	27.55 ± 0.90 a
N144 + HC1.5%	99.52 ± 5.03 a	117.90 ± 3.92 ab	30 ± 1 ab	138 ± 11 a	29.45 ± 1.41 b

Note: Data are shown as mean ± SD (*n* = 3). Different lowercase letters indicate significant differences between treatments, according to Duncan’s multiple range test (*p* < 0.05).

**Table 2:** Effects of applying different amounts of hydrochar (HC) to rice paddy soil with different nitrogen (N) inputs on the N uptake capacity of rice.

Treatment	Nitrogen Uptake of Straw (mg/pot)	Nitrogen Uptake of Grain (mg/pot)	Total Nitrogen Uptake (mg/pot)
N240	2.62 ± 0.15 ab	12.54 ± 1.03 a	15.16 ± 1.12 a
N240 + HC0.5%	2.45 ± 0.11 ab	12.15 ± 1.94 a	14.60 ± 1.98 a
N240 + HC1.5%	2.59 ± 0.14 ab	13.17 ± 0.39 a	15.76 ± 0.41 a
N192	2.24 ± 0.26 a	13.36 ± 0.31 a	15.61 ± 0.34 a
N192 + HC0.5%	2.98 ± 0.22 b	11.89 ± 0.33 a	14.88 ± 0.29 a
N192 + HC1.5%	5.99 ± 0.52 e	12.49 ± 0.94 a	18.47 ± 1.47 a
N144	4.10 ± 0.58 c	11.15 ± 0.56 a	15.26 ± 1.06 a
N144 + HC0.5%	4.80 ± 0.19 d	12.69 ± 0.78 a	17.49 ± 0.58 a
N144 + HC1.5%	3.04 ± 0.33 b	10.60 ± 3.51 a	13.64 ± 3.31 a

Note: Data are shown as mean ± SD (*n* = 3). Different lowercase letters indicate significant differences between treatments, according to Duncan’s multiple range test (*p* < 0.05).

## Data Availability

All data are included in the main text.
